# Time Perspective Matters for Working Alliance in Schizophrenia Care: Insights From the DiAPAson Project

**DOI:** 10.1002/cpp.70151

**Published:** 2025-09-17

**Authors:** Cristina Zarbo, Maciej Stolarski, Donato Martella, Elena Toffol, Giovanni de Girolamo, for the DIAPASON consortium

**Affiliations:** ^1^ Department of Psychology University of Milano‐Bicocca Milan Italy; ^2^ Faculty of Psychology University of Warsaw Poland; ^3^ Unit of Epidemiological Psychiatry and Digital Mental Health IRCCS Istituto Centro san Giovanni di Dio Fatebenefratelli Brescia Italy; ^4^ Department of Statistics and Quantitative Methods University of Milano‐Bicocca Milan Italy

**Keywords:** interpersonal skills, present fatalistic, schizophrenia, time perspective, treatment, working Alliance

## Abstract

**Objectives:**

The time perspective dimensions and balanced time perspective (BTP) may play a crucial role in the therapeutic process of individuals with schizophrenia spectrum disorders (SSDs). This study aimed to investigate the relationship between BTP, working alliance (WA) and psychiatric symptomatology in individuals with SSD.

**Methods:**

A total of 313 residential patients with SSD were recruited from 98 residential facilities in Italy. Clinicians completed the Brief Psychiatric Rating Scale (BPRS) and the Brief Negative Symptom Scale (BNSS), while patients completed the Zimbardo Time Perspective Inventory (ZTPI) and the Working Alliance Inventory–Short Form (WAI). Deviation from Balanced Time Perspective–revised (DBTP‐r) was applied as an estimate of unbalanced TP. Statistical analyses included Pearson correlations and mediation models. Age, sex, illness duration and years of education were controlled in mediation analyses.

**Results:**

DBTP‐r showed significant negative correlations with WAI total and positive correlations with BPRS and BNSS. ZTPI‐Present Fatalistic negatively correlated with WAI‐Goal. The WAI partially mediated the effect of DBTP‐r on both BPRS and BNSS, while DBTP‐r partially mediated the impact of WAI on both BPRS and BNSS.

**Conclusions:**

BTP played a dual role, acting both as a contributing factor to and a result of the WA. The present‐fatalistic TP emerged as a key factor in shaping WA concerning therapeutic goals. Findings underscore the importance of considering (B)TP as both a potential resource and a therapeutic target in treating SSD.

## Summary

- A balanced time perspective and the quality of the working alliance play a key role in influencing psychiatric outcomes among individuals with schizophrenia spectrum disorders (SSD).
- Individuals with SSD who demonstrate a more balanced time perspective tend to establish stronger working alliances with their treating staff.
- Conversely, higher levels of working alliance with the treating staff may also promote a more balanced time perspective in individuals with SSD.
- A predominant present‐fatalistic orientation may act as a barrier to the development of an effective working alliance in this population.

## 1 Introduction

Humans have developed a unique capacity for mental time travel—that is, the ability to reconstruct events from the personal past and to imagine possible future scenarios (Suddendorf and Corballis 2007). This ability has enabled a variety of essential adaptations, including counterfactual thinking (Terrett et al. 2016), planning and goal setting (Klein et al. 2010), intertemporal choice and delay of gratification (Bulley et al. 2016), as well as deliberate practice (Suddendorf et al. 2015).

People manifest relatively stable individual differences in tendencies and biases associated with mental time travel. The theory of time perspective (Zimbardo and Boyd 1999; Stolarski et al. 2018) provides a vital framework for analysing individual differences in how people temporally frame their personal experience, along with its consequences for emotional, cognitive and behavioural outcomes. In Zimbardo and Boy's (1999) conceptualization, the time perspective universe comprises the following five dimensions: past positive, past negative, present fatalistic (PF), present hedonistic and future (F). Each reflects a distinct tendency to focus on a specific temporal horizon (e.g., past) in a particular evaluative manner (e.g., positive) and is operationalized as a trait‐like dimension.

Zimbardo and Boyd (1999) have noted that being overly concentrated on any temporal horizon is maladaptive: to effectively adapt to various life situations one needs to be able to find a proper balance between the past, the present and the future. Such temporal plasticity, depicted in the concept of balanced time perspective (BTP), allows for flexible shifting between these temporal dimensions based on situational demands and has vital consequences for various features of socioemotional adaptation (Stolarski et al. 2020) and mental health (Stolarski, Czajkowska‐Łukasiewicz, et al. 2024).

The concept of mental time travel may be particularly relevant for understanding the role of time perspective in therapeutic processes. Mental time travel provides the cognitive foundation for forming and maintaining a BTP: the ability to flexibly recall meaningful past experiences, remain engaged in the present, and project oneself into the future in a constructive way. Deficits in mental time travel have been documented in schizophrenia, including difficulties in generating specific autobiographical memories and imagining detailed future scenarios (D'Argembeau et al. 2008; Raffard et al. 2010). These impairments may reduce temporal flexibility and foster maladaptive orientations such as a PF or past‐negative focus, thereby contributing to deviations from a BTP. In turn, such limitations may undermine essential relational processes, as individuals struggle to build trust on the basis of coherent past experiences or to engage in goal‐oriented collaboration grounded in a shared vision of the future.

BTP may be particularly relevant in clinical settings, especially for individuals with schizophrenia spectrum disorders (SSDs), where building a working alliance (WA) can be challenging. Many individuals with SSD have experienced early trauma affecting their adult relationships (Varese et al. 2012), and poor engagement in treatment is a key barrier to successful outcomes, impacting symptom severity, medication adherence, dropout rates and hospitalisation (McCabe et al. 2012; Priebe et al. 2011): For all these reasons, establishing a good WA with these patients is particularly important. The concept of WA refers to the relationship between a therapist and a patient, and its ability to facilitate change during therapy (Horvath and Symonds 1991). The most widely accepted operational model of WA is Bordin's three‐dimensional model, which states that a positive WA is achieved through agreement on 1) treatment goals, 2) tasks and 3) the development of a strong bond between the therapist and patient (Bordin 1979; Doran et al. 2017).

Research suggests that individuals with SSD exhibit pronounced deviations from the BTP (DBTP), characterised by a stronger past‐negative and PF orientation and a weaker past‐positive and future perspective (Stolarski, Czajkowska‐Łukasiewicz, et al. 2024). Such an imbalance is linked to greater symptom severity, impaired working memory and reduced social and daily functioning (Chen et al. 2023; Damiani et al. 2023; Zarbo et al. 2023). Given the impact of time perspective on interpersonal skills (including metallisation, trust, attachment and social functioning), its role in therapeutic relationships warrants investigation. A recent study (Winquist and Rönnlund 2024) found that an unbalanced time perspective correlates with hypomentalisation, a cognitive deficit impairing the ability to infer others' thoughts, emotions and intentions. This limitation may hinder social interactions and the formation of a strong WA, which is crucial for treatment success (Lüdemann et al. 2021). Interpersonal trust, attachment and social functioning are also closely tied to time perspective. Individuals with BTP tend to have greater interpersonal trust, as they hold fewer negative and more positive past relational experiences (see Stolarski et al. 2020, for a review), with possible positive outcomes within the therapeutic process. Moreover, those with higher BTP develop more fulfilling relationships, a trend initially observed in romantic partnerships (Stolarski et al. 2016) but supported by broader research linking BTP to better social functioning (Holman and Zimbardo 2009), higher emotional intelligence (Stolarski et al. 2011) and lower social anxiety (Stout et al. 2024). Additionally, poorer temporal balance is associated with less secure attachment patterns (Akirmak 2014), which may weaken the WA.

Given that individuals with SSD often struggle with interpersonal skills (Chung et al. 2014; Fett et al. 2012), and considering the recently established link between BTP and interpersonal abilities (Winquist and Rönnlund 2024; Holman and Zimbardo 2009; Stout et al. 2024; Akirmak 2014), we hypothesize that higher levels of DBTP in individuals with SSD residing in residential facilities (RFs) are associated with a weaker WA and greater psychiatric symptomatology. Moreover, BTP may serve both as a predictor and an outcome of WA. The self‐regulatory features of BTP (Stolarski et al. 2018) can foster interpersonal trust and support the development of deeper and more stable interpersonal bonds, which underpin strong therapeutic alliances. Conversely, the therapeutic functions of a well‐established alliance may promote temporal balance—by providing positive personal experiences that reshape perceptions of the past or reduce anxiety about the future. As a result, this feedback loop may become an integral part of the therapeutic process, illustrating the mechanisms underlying the effects of both WA and BTP on psychiatric symptoms (Damiani et al. 2023; Fusar‐Poli et al. 2024; Iovoli et al. 2024; Shattock et al. 2018; Stolarski, Czajkowska‐Łukasiewicz, et al. 2024). To test these predictions, our study examines the relationships between BTP, WA with staff and psychiatric severity in individuals with SSD residing in RFs. Specifically, we explore (1) the associations between TP dimensions, BTP and WA; (2) the mediating role of WA in the relationship between BTP and psychiatric outcomes; (3) the mediating role of BTP in the relationship between WA and psychiatric outcomes; (4) based on aim 1's findings, the mediating role of WA in the link between the PF dimension of time perspective and psychiatric outcomes. By investigating these associations, we aim to clarify how temporal perspective may influence treatment engagement and clinical outcomes among participants with SSD, offering new insights into improving therapeutic relationships and interventions.

## 2 Methods

From October 2020 to October 2021, 313 residential patients with SSD were recruited from 98 RFs in Italy as part of the DiAPAson project (de Girolamo et al. 2020). On average, each RF housed 12.8 (±5.7) residents and recruited approximately 3.3 (±2.6) patients, representing roughly 25% of the facility's residents.

We included patients with an SSD diagnosis according to the Diagnostic and Statistical Manual of Mental Disorders, Fifth Edition (DSM‐5) criteria (American Psychiatric Association (APA) 2013), who were between the ages of 18 and 55, able to talk and write in Italian and in treatment at RFs. We excluded patients who were unable to provide informed consent or who reported severe cognitive deficits, a recent (last 6 months) diagnosis of substance use disorder according to the DSM‐5 criteria (American Psychiatric Association (APA) 2013) or a history of clinically significant head injury or cerebrovascular/neurological disease.

Local ethical committees approved the study (see the specific section below). All participants provided informed consent for their participation.

### 2.1 Recruitment

At each study centre, treating clinicians invited participants under their care to participate in the study. The participants were provided with detailed information about the study and had the opportunity to ask questions. Residential patients were recruited using an alphabetical list of patients with SSD present on an index day prepared by the facility chiefs; based on this list, residential patients were consecutively invited to participate in the study until the recruitment target was achieved.

Some of the assessment tools were completed by the treating clinician, while Research Assistants helped the participants complete self‐reported questionnaires if needed. All measures were completed using the same methodology to make the results comparable and reduce any potential bias. Standardized clinical measures were used to collect clinical data to minimise methodological biases.

### 2.2 Clinical Assessments

Psychiatric history was assessed using a structured ad hoc survey aimed at collecting information such as the current diagnosis, illness duration, current medication and lifetime duration of psychiatric hospitalization. Each treating clinician completed the psychiatric survey, the Brief Psychiatric Rating Scale (BPRS) and the Brief Negative Symptom Scale (BNSS). The 24‐item BPRS (Morosini and Casacchia 1995; Overall and Gorham 1962) was used to assess the presence and severity of psychopathology. The BPRS items were rated on a 7‐point scale ranging from 1 (not present) to 7 (extremely severe). Negative symptoms severity was assessed using the BNSS (Mucci et al. 2015; Strauss et al. 2012), a 13‐item instrument that evaluates blunted affect, alogia, asociality, anhedonia and avolition (from 0 —*not present*—to 6—*severe deficit*). A higher total score on both BPRS and BNSS indicates a higher symptom severity. In the present study, both BPRS and BNSS demonstrated good internal consistency (BPRS: Cronbach α = 0.807, BNSS: α = 0.935).

### 2.3 Self‐Reported Assessment

An Italian Translation of the Zimbardo Time Perspective Inventory (ZTPI; Zimbardo and Boyd 1999) was used to measure individual differences in TP. The scale includes 56 items and five subscales, which are past positive (PP), past negative (PN), present hedonistic (PH), present fatalistic (PF) and Future (F). ZTPI items were rated on a 5‐point Likert scale ranging from very uncharacteristic (1) to very characteristic (5) of the respondent. The composition of the scores across the five dimensions of ZTPI allowed for the assessment of the adaptiveness of the temporal profile of the individual. Zimbardo and Boniwell (2004) proposed that the most functional profile is reflected in low scores on maladaptive orientations (PN and PF), high scores on functional ones (PP and F) and a moderate score on the remaining PH orientation. The method proposed by Stolarski et al. (2011), referred to as the DBTP and its revised version (DBTP‐r) endorsed by Jankowski et al. (2020), allows the assessment of the fit between the individual's time perspective profile and the optimal profile. The DBPT‐r is defined as the Euclidean distance between the optimal and the empirical levels of time perspective: $\mathrm{DBT}P_{r}=\sqrt{{\left(1-\mathrm{PN}\right)}^{2}+{\left(5-\mathrm{PP}\right)}^{2}+{\left(1-\mathrm{PF}\right)}^{2}+{\left(3.4-\mathrm{PH}\right)}^{2}+{\left(5-F\right)}^{2}}$. A value close to zero indicates an almost perfectly balanced TP (the theoretical ideal), whereas a large positive value indicates an unbalanced time perspective profile. The ZTPI subscale showed acceptable to good internal consistency in our sample (ZTPI‐PN: α = 0.784, ZTPI‐PH: α = 0.731, ZTPI‐F: α = 0.631, ZTPI‐PP: α = 0.712, ZTPI‐PF: α = 0.725).

WA was assessed through the WA Inventory short‐form (WAI‐SF) (Lingiardi 2002). The WAI‐SF includes 12 items that can be rated on a 7‐point Likert scale, from 1 (‘*never*’) to 7 (‘*always*’). The total score can range between 12 and 84, with higher scores indicating a greater WA. Similarly, to the original 36‐item WAI (Horvath and Greenberg 1989), three subscales measuring the three domains of WA can be computed: (a) agreement between patient and therapist about treatment goals (Goals); (b) agreement between patient and therapist on tasks to achieve these goals (Task); and (c) quality of the bond between patient and therapist (Bond). Patients with SSD referred to the WA with a specific staff member with whom he/she had a “one‐to‐one” therapeutic relationship (i.e., nurse, psychiatrist, clinical psychologist, health assistant, educator or occupational therapist). The WAI demonstrated high internal consistency (WAI total: α = 0.894, WAI‐Goal: α = 0.690, WAI‐Task: α = 0.789, WAI‐Bond: α = 0.834).

### 2.4 Statistical Analyses

The data are presented as mean ± standard deviation (SD), median and range or count and percentage for categorical variables. First, Pearson correlation coefficients were calculated to assess relationships among the five ZTPI subscales, DBTP‐r, the three WAI subscales, the WAI total score and psychiatric symptoms (BPRS and BNSS). Multiple comparisons were adjusted using the Holm–Bonferroni method. To further explore the interplay between time perspective, WA and clinical outcomes, a series of mediation analyses were conducted using 5000 bootstrap iterations to estimate total, direct and indirect effects. All models controlled for age, sex, illness duration and years of education. The first set of mediation models examined whether WAI mediated the relationship between DBTP‐r and psychiatric symptom severity (BPRS and BNSS). A complementary set tested the reverse direction, with WAI as the independent variable and DBTP‐r as the mediator, while keeping BPRS and BNSS as outcome variables. Additionally, based on the results obtained with correlation analyses, we explored the specific role of the ZTPI‐PF subscale. In particular, we tested whether the WAI‐goal subscale mediated the relationship between ZTPI‐PF and BPRS and BNSS. All analyses were conducted using R software (version 4.3.2), with statistical significance set at *p* < 0.05.

## 3 Results

### 3.1 Sociodemographic and Clinical Characteristics of the Sample

Table 1 presents the demographic and clinical characteristics of the participants (*N* = 301). The average age was 41.3 years (SD = 10.0), with over 70% being male. Participants had a mean education level of 11.5 years (SD = 3.2). The average illness duration was 18.4 years (SD = 9.8). Means and standard deviations of all measures are included in Table 1.

**TABLE 1:** Sociodemographic, clinical and psychological characteristics of the sample (*N* = 301).

Variables	
**Age**	
*Mean (SD)*	41.3 (10.0)
*Median (range)*	43.0 (33.0–50.0)
**Sex**	
*Male, n (%)*	211 (70.1%)
*Female, n (%)*	90 (29.9%)
**Illness duration (years)**	
*Mean (SD)*	18.4 (9.8)
*Median (range)*	20.0 (10.0–25.0)
**Education (years)**	
*Mean (SD)*	11.5 (3.2)
*Median (range)*	12.0 (8.0–13.0)
**BPRS total score**	
*Mean (SD)*	50.9 (16.4)
*Median (range)*	49.0 (38.0–60.0)
**BNSS total score**	
*Mean (SD)*	26.0 (16.5)
*Median (range)*	24.0 (13.0–37.0)
**WAI total score**	
*Mean (SD)*	62.1 (13.7)
*Median (range)*	64.0 (54.0–72.0)
**WAI‐Goal**	
*Mean (SD)*	20.6 (5.0)
*Median (range)*	21.0 (18.0–24.0)
**WAI‐Task**	
*Mean (SD)*	20.9 (5.1)
*Median (range)*	21.0 (18.0–25.0)
**WAI‐Bond**	
*Mean (SD)*	20.6 (5.5)
*Median (range)*	21.0 (18.0–25.0)
**ZTPI‐PP**	
*Mean (SD)*	3.1 (0.7)
*Median (range)*	3.1 (2.7–3.6)
**ZTPI‐PN**	
*Mean (SD)*	3.5 (0.7)
*Median (range)*	3.5 (3.1–4.0)
ZTPI**‐PF**	
*Mean (SD)*	2.9 (0.7)
*Median (range)*	3.0 (2.4–3.4)
**ZTPI‐PH**	
*Mean (SD)*	3.2 (0.5)
*Median (range)*	3.2 (2.9–3.5)
**ZTPI‐F**	
*Mean (SD)*	3.2 (0.5)
*Median (range)*	3.2 (2.9–3.5)
**DBTP‐r**	
*Mean (SD)*	4.3 (0.8)
*Median (range)*	4.3 (3.7–4.8)

Abbreviations: BPRS, Brief Psychiatric Rating Scale; BNSS, Brief Negative Symptom Scale; WAI, WA Inventory; ZTPI, Zimbardo Time Perspective Inventory; ZTPI‐F, future; ZTPI‐PH, present hedonistic; ZTPI‐PF, present fatalistic; ZTPI‐PN, past negative; ZTPI‐PP, past positive.

### 3.2 Correlations Between WA, Time Perspective and Psychiatric Symptoms

**FIGURE 1:**
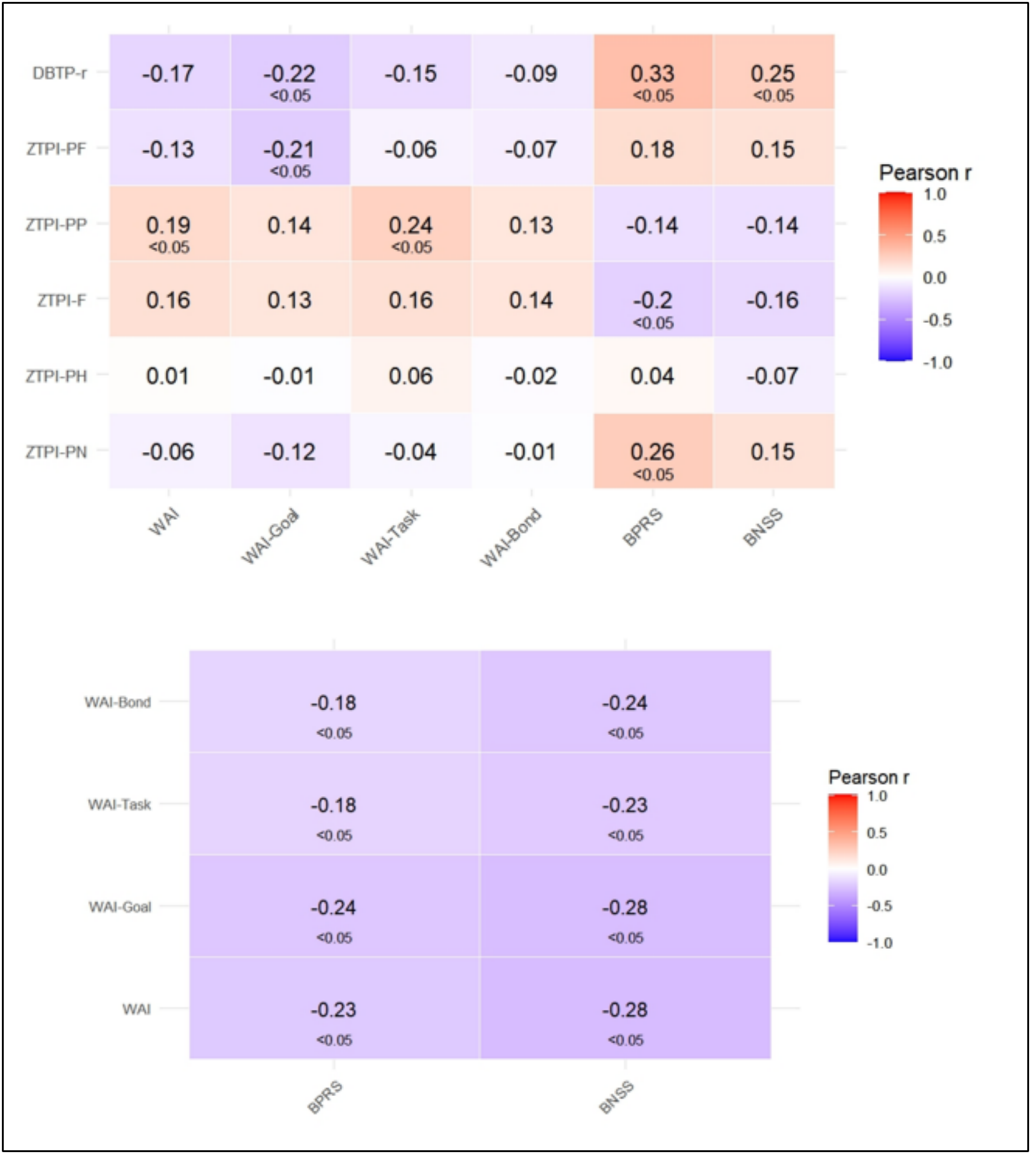
Correlation analysis of ZTPI subscales, DBTP‐r, WAI and psychiatric symptoms (BPRS, BNSS).

Figure 1 illustrates Pearson correlation coefficients between ZTPI subscales, DBTP‐r, WAI subscales, the WAI total score and psychiatric symptoms (BPRS and BNSS). The colour gradient represents the strength and direction of correlations, with red indicating positive correlations and blue indicating negative correlations. DBTP‐r was negatively correlated with WAI‐Goal (*r* = −0.22) and WAI total (r = −0.17) scores and positively associated with BPRS (*r* = 0.33) and BNSS (*r* = 0.25). ZTPI‐PF negatively correlated with WAI‐Goal (*r* = −0.21), while ZTPI‐PP positively correlated with WAI total (*r* = 0.19) and WAI‐Task (*r* = 0.24) scores. All WAI subscales showed significant negative correlations with clinical measures. BPRS negatively correlated with WAI‐Bond (*r* = −0.18), WAI‐Task (*r* = −0.18), WAI‐Goal (*r* = −0.24) and WAI total (*r* = −0.23); BNSS negatively correlated with WAI‐Bond (*r* = −0.24), WAI‐Task (*r* = −0.23), WAI‐Goal (*r* = −0.28) and WAI total (*r* = −0.28).

### 3.3 The Mediating Role of WA in the Relationship Between BTP and Psychiatric Symptoms

**FIGURE 2:**
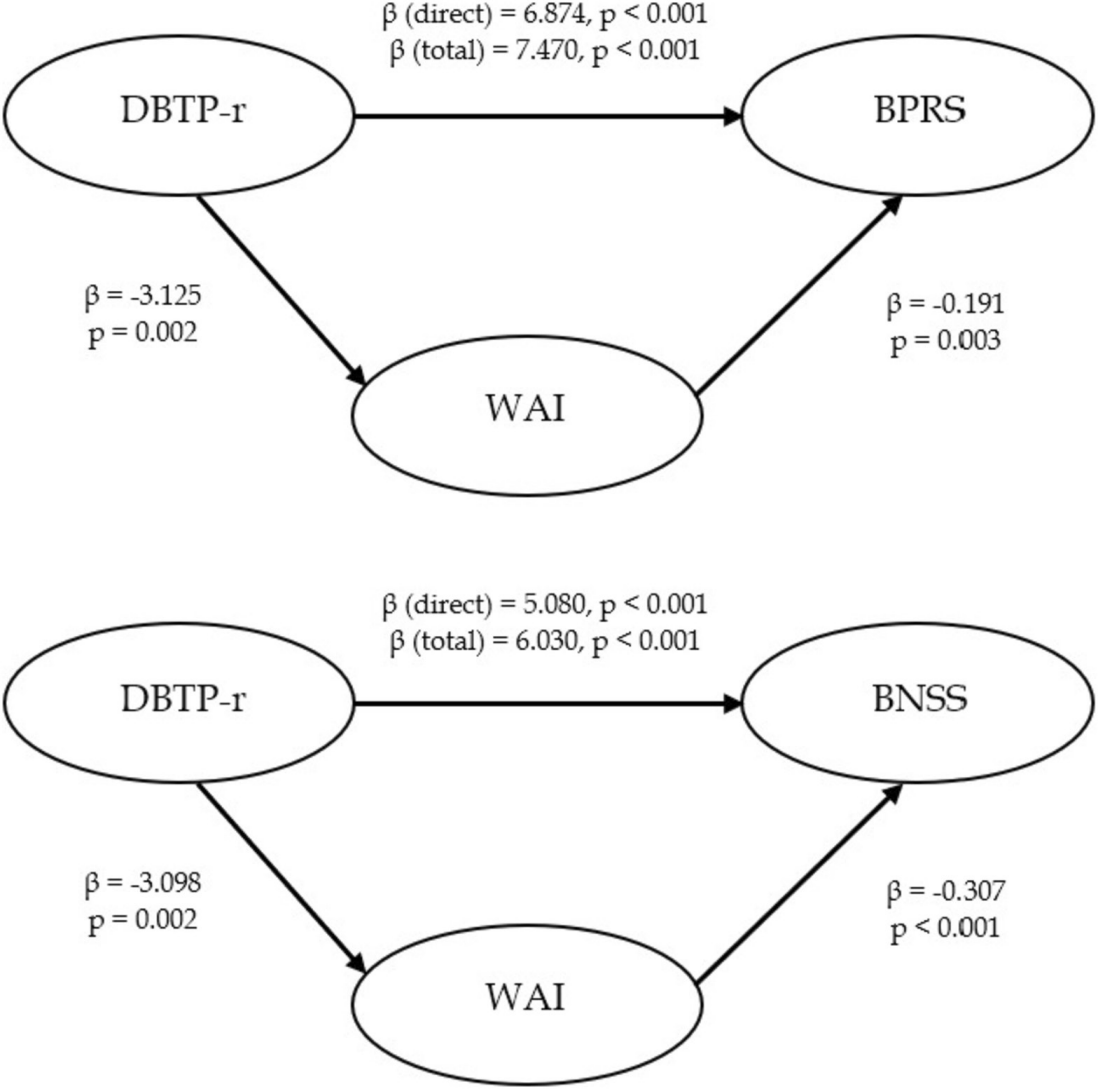
Mediating role of Working Alliance (WAI–total score) between Time Perspective (DBTP‐r) and psychiatric symptoms (BPRS; BNSS).

Figure 2 illustrates two mediation models examining the mediating role of the WAI in the relationship between DBTP‐r and psychiatric symptom severity (BPRS and BNSS).

In the first model, a higher score on the DBTP‐r was associated with higher BPRS scores. The total effect of DBTP‐r on BPRS was significant (β total = 7.470, *p* < 0.001). When controlling for WAI, the direct effect remained significant but was slightly reduced (β direct = 6.874, *p* < 0.001).

The second model shows a similar pattern for BNSS scores (β direct = 5.080, *p* < 0.001; β total = 6.030, *p* < 0.001). While WAI mediated the relationship by improving BNSS scores, the direct effect of DBTP‐r remained predominant. Overall, these findings suggest that the effect of higher DBTP‐r on worse psychiatric symptom severity is partially mediated by reduced WA. The ratio of indirect to total effect was approximately 8.7% for BPRS and 18.7% for BNSS, suggesting modest mediation effects in both models.

### 3.4 The Mediating Role of BTP in the Relationship Between WA and Psychiatric Symptoms

**FIGURE 3:**
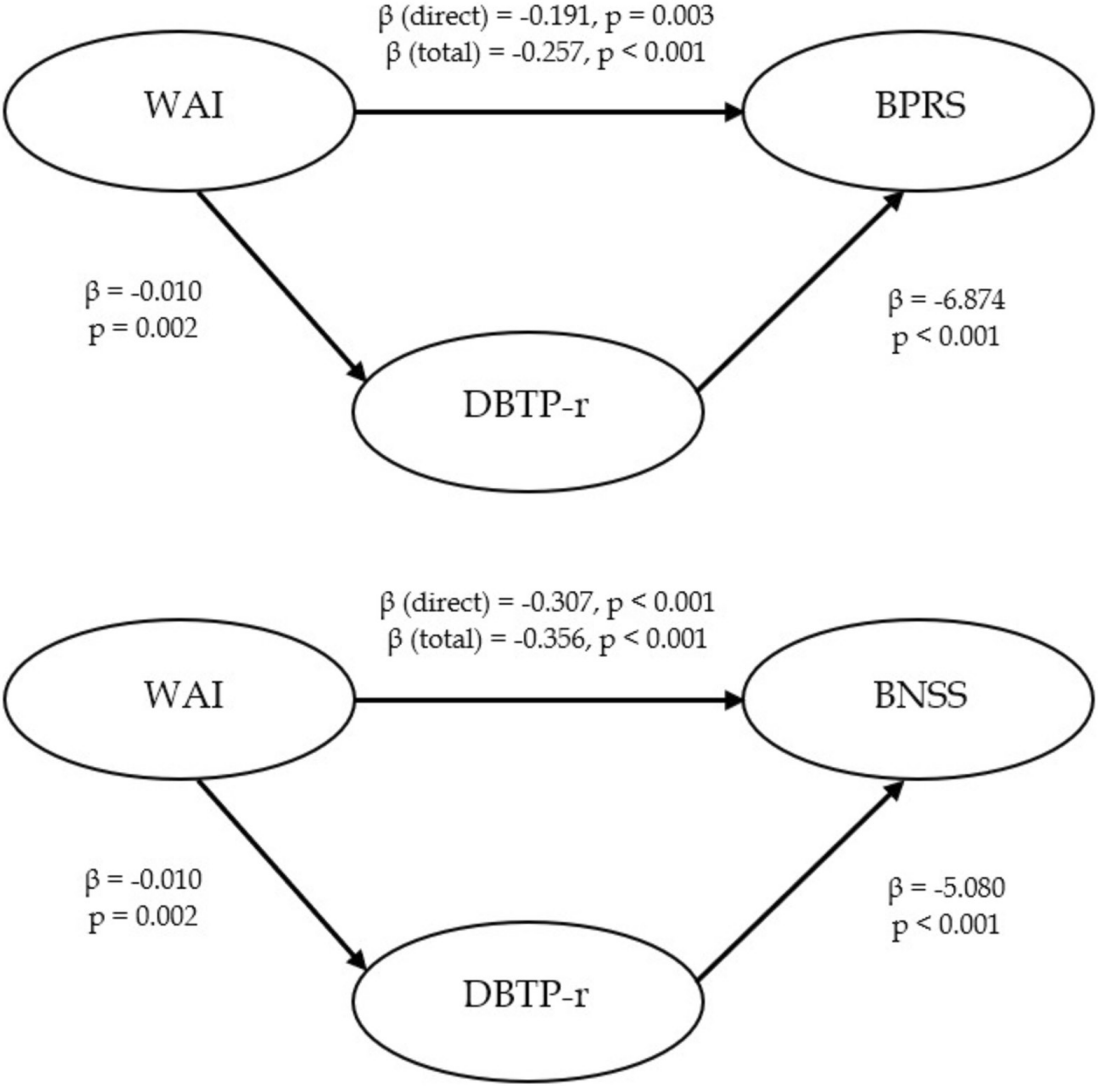
Mediating role of Time Perspective (DBTP‐r) between Working Alliance (WAI–total score) and psychiatric symptoms (BPRS; BNSS).

Figure 3 illustrates two mediation models examining the role of DBTP‐r in the relationship between WAI and psychiatric symptom severity (BPRS and BNSS).

For BPRS, the total effect of WAI on symptom severity was significant (β total = −0.257, *p* < 0.001). When accounting for DBTP‐r, the direct effect of WAI remained significant, though slightly reduced (β_direct = −0.191, *p* = 0.003), indicating partial mediation, with DBTP‐r serving as a significant mediator. For BNSS, a similar pattern emerged, with WAI exerting both direct and indirect negative effects (β direct = −0.307, *p* < 0.001; β total = −0.356, *p* < 0.001), again mediated by DBTP‐r. Overall, these findings suggest that the effect of higher WAI on lower psychiatric symptom severity is partially mediated by reduced BTP. In particular, the indirect to total effect ratio was approximately 36.0% for BPRS and 16.5% for BNSS, indicating a stronger mediation pathway in the case of BPRS.

### 3.5 The Mediation Role of the WA About Goals in the Relationship Between PF and Psychiatric Symptoms

**FIGURE 4:**
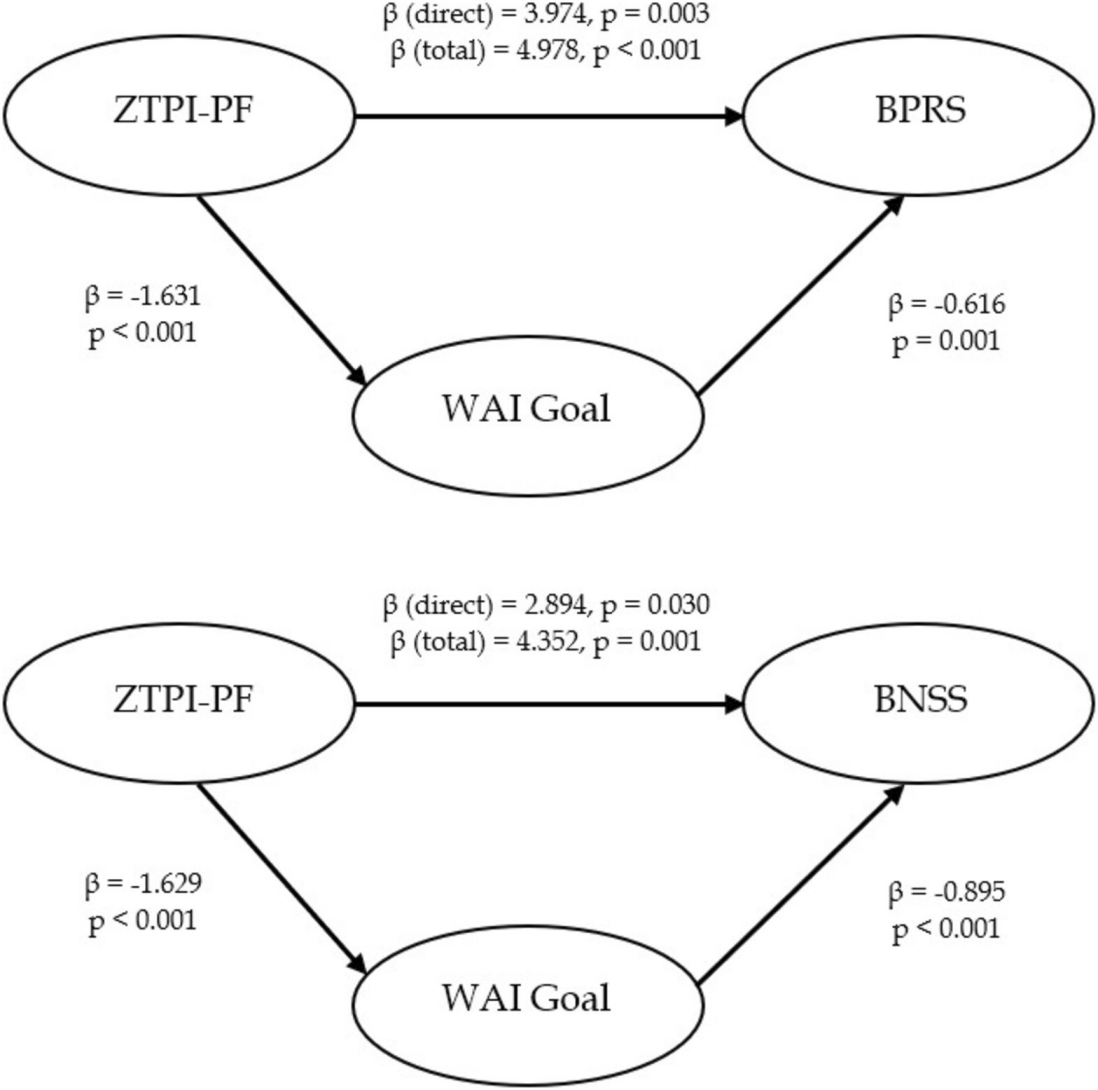
Mediating role of Working Alliance on Therapeutic Goals (WAI‐Goal) Time Perspective‐Present Fatalistic Dimension (ZTPI‐PF) and psychiatric symptoms (BPRS; BNSS).

The pattern of associations observed in the correlational analysis (see Figure 1) suggests that the relationship between PF time perspective and symptom severity may be mediated by WA. Figure 4 illustrates two mediation models examining the role of WAI‐Goal in the relationship between ZTPI‐PF and psychiatric symptom severity (BPRS and BNSS).

For BPRS, ZTPI‐PF had a significant total effect on symptom severity (β total = 4.978, *p* < 0.001) and also a direct effect (β_direct = 3.974, *p* = 0.003), with WAI‐Goal serving as a significant mediator. Specifically, ZTPI‐PF negatively impacted WAI‐Goal, which in turn was negatively associated with BPRS scores. For BNSS, a similar pattern emerged, with ZTPI‐PF exerting both direct and indirect effects (β direct = 2.894, *p* = 0.030; β total = 4.352, *p* = 0.001), again mediated by WAI‐Goal. ZTPI‐PF negatively influences WAI‐Goal, which was negatively correlated with BNSS scores. These findings suggest that the effect of a higher PF perspective on psychiatric symptom severity is partially mediated by reduced WA with the treating staff. The indirect to total effect ratio was approximately 25.3% for BPRS and 50.4% for BNSS, indicating that the mediating role of WAI‐Goal was particularly significant regarding negative symptoms.

## 4 Discussion

This study explored the relationships between time perspective, WA and psychiatric outcomes in 301 individuals with SSD residing in RFs in Italy, while controlling for age, sex, illness duration and years of education. Our findings revealed that both BTP and WA were significant predictors of psychiatric outcomes. The interplay between these two variables may be complex: In the two alternative mediation analyses, BTP displayed significant effects in two roles, functioning as both a contributing factor to and an outcome of the WA—influencing the therapeutic relationship while also being shaped by it. Moreover, the PF dimension of time perspective emerged as a key factor in shaping WA, particularly regarding therapeutic goals. These interconnected factors directly and indirectly influence psychiatric outcomes, impacting both positive and negative symptomatology.

Surprisingly, the past‐negative time perspective—typically showing the most consistent and replicable effects on various clinical symptoms (Stolarski, Czajkowska‐Łukasiewicz, et al. 2024)—did not prove meaningful for any of the measured forms of working alliance. Although its effects on SSD symptomatology were replicated in the present study (see Figure 1), these associations appear to stem from more fundamental mechanisms (e.g., genetic or temperamental factors; see Stolarski, Zawadzki, et al. 2024) rather than alliance‐related features.

### 4.1 Time Perspective, Especially a Focus on the Present Fatalistic Dimension, Affects WA With the Treating Staff

Our findings highlight the important role of time perspective in shaping the WA between individuals with SSD and their treating staff. Indeed, individuals with a more BTP may find it easier to develop well‐functioning alliances with the treating staff.

These results seem to align with previous research illustrating the regulatory role of a BTP in interpersonal contexts, including more adaptive attachment patterns, greater emotional intelligence, improved mentalisation and higher relationship quality (Laghi et al. 2009; Stolarski et al. 2011; Winquist and Rönnlund 2024).

More specifically, the PF dimension significantly influenced the WA, particularly in reaching agreement on therapeutic goals. The way individuals perceive and relate to time profoundly affects their behaviours and interpersonal relationships, including therapeutic interactions. This is particularly relevant for individuals with an unbalanced time perspective, who may struggle with understanding complex mental processes (Winquist and Rönnlund 2024), display lower interpersonal trust due to a more negative representation of the past (Laghi et al. 2009), and exhibit less secure attachment patterns (Akirmak 2014). It is therefore plausible that interpersonal skills—such as trust, attachment, and mentalisation—mediate the relationship between BTP and the WA. Moreover, individuals with SSD who exhibit stronger fatalistic, helpless and hopeless attitudes toward life and the future tend to experience more severe psychiatric symptoms. This may, in part, be due to difficulties in establishing a strong WA, particularly in negotiating therapeutic goals.

Our findings are consistent with previous research showing that individuals with a dominant fatalistic orientation tend to have lower motivation (Zimbardo and Boyd 1999) and reduced goal‐directed behaviour (Baird et al. 2021). This presents a significant challenge in clinical settings, as agreement on treatment goals is a key component of a strong WA (Horvath and Greenberg 1989) and is crucial for positive therapeutic outcomes.

### 4.2 WA With the Treating Staff May Enhance a BTP

Our findings suggest that a strong WA may contribute to a more BTP, which in turn is associated with better psychiatric outcomes. The therapeutic alliance is built on trust, relationship‐building and the development of a supportive interpersonal bond, which can have a reparative effect for individuals with a history of dysfunctional relationships (Fonagy and Allison 2014). By fostering mentalisation, trust and emotional engagement, a strong alliance may help mitigate a past‐negative orientation and reduce fatalistic tendencies, ultimately enhancing future‐oriented thinking.

## 5 Conclusions

These findings highlight the importance of time perspectives both as a resource and a therapeutic target in the treatment of SSD. The relationship between BTP and WA may be reciprocal, where a change in one of these variables could lead to a positive feedback loop, promoting desirable outcomes in a clinical setting. Addressing time perspective imbalances, particularly PF orientations, may strengthen the WA and improve psychiatric outcomes. Given the effects of BTP and PF on treatment engagement, clinicians should carefully assess patients' time perspectives and incorporate temporal balance as a treatment goal. Encouraging future‐oriented thinking and reducing fatalistic attitudes may enhance therapy engagement, leading to better clinical outcomes. Future research should explore longitudinal interventions aimed at modifying time perspective in clinical populations, assessing their impact on treatment engagement and overall well‐being.

While this study provides valuable insights into the relationships between time perspective, WA and psychiatric outcomes, it has some limitations. The cross‐sectional design prevents any definite causal inferences regarding the directionality of these associations. Additionally, we did not account for potential differences in the role of specific therapeutic figures (e.g., psychiatrists, psychologists, nurses) in shaping the WA. Future studies should employ longitudinal designs and consider the influence of different treating professionals on time perspective and therapeutic engagement.

## Author Contributions

**Cristina Zarbo:** conceptualisation, data curation, investigation, methodology, project administration, supervision, supervision, writing – original draft preparation, writing – review and editing. **Maciej Stolarski:** conceptualisation, methodology, writing – original draft preparation, writing – review and editing. **Donato Martella:** conceptualisation, data curation, formal analysis, visualisation, writing – original draft preparation, writing – review and editing. **Elena Toffol:** conceptualisation, methodology, writing – original draft preparation, writing – review and editing. **Giovanni de Girolamo:** conceptualization, funding acquisition, methodology, project administration, resources, supervision, writing – original draft preparation, writing – review and editing.

## Ethics Statement

The study has been approved by the ethical committees (ECs) of the three main participating centres: EC of IRCCS Istituto Centro San Giovanni di Dio Fatebenefratelli (July 31, 2019; No. 211/2019), EC of Area Vasta Emilia Nord (September 25, 2019; No. 0025975/19) and EC of Pavia (September 02, 2019, No. 20190075685); Ethical Approval was also obtained from ECs of all participating centres.

## Conflicts of Interest

The authors declare no conflicts of interest.

## Data Availability

The data that support the findings of this study are openly available in Zenodo at https://zenodo.org/records/14966495, reference number 10.5281/zenodo.14966495.
